# Failed primary percutaneous coronary intervention in a middle-aged man without cardiovascular risk factors: left atrium myxoma

**DOI:** 10.11604/pamj.2020.36.6.14845

**Published:** 2020-05-06

**Authors:** Nahid Azdaki, Seyedali Moezi, Mahmood Hosseinzadehmaleki, Marjan Farzad

**Affiliations:** 1 Cardiovascular Diseases Research Center, Department of Cardiology, School of Medicine, Birjand University of Medical Sciences, Birjand, Iran; 2 Razi Clinical Research Development Unit (RCRDU), Birjand University of Medical Sciences, Birjand, Iran; 3 Cardiovascular Diseases Research Center, Birjand University of Medical Sciences, Birjand, Iran

**Keywords:** Cardiac myxoma, embolism, left atrium myxoma

## Abstract

Embolic events are rare presentation of myxoma, which is one of the most prevalent benign cardiac tumors. Here we report the case of a 53-year-old man with presentation of acute anterior infarction and occlusion of the left anterior descending artery in association with left atrial myxoma. Intracoronary aspiration thrombectomy along with frequent balloon inflation was failed to recover distal coronary blood flow.

## Introduction

Cardiac myxomas are the most common benign cardiac neoplasm. The exact etiology of the tumor has yet to be fully determined. Hemodynamic instability, intermittent syncope, congestive heart failure, pulmonary hypertension, sudden death and systemic embolization are some presentations of myxoma [1-3]. Cardiac myxoma more commonly affects the atria than the ventricles [4]. Atrial myxomas are associated with systemic embolization in 30 to 40% of cases, however, embolic coronary manifestations are rare and reported with 0.06% prevalence which can be due to the fragmentation of the tumor mass or embolization of thrombotic aggregation at the surface of the mass [5]. As Coronary embolisms are themselves the rare etiologies of the non-sclerotic coronary artery diseases (NA-CAD). ST elevation myocardial infarction (STEMI) occurs rarely by the left atrium myxoma [6]. In this regard, we report the case of a 53-year-old man with presentation of acute anterior infarction and occlusion of the left anterior descending artery, which transthoracic echocardiography revealed surprisingly a myxoma in the left atrium.

## Patient and observation

A 53-year-old man referred to emergency department with typical angina. Neither history of heart diseases in his past medical history nor any risk factors of cardiac diseases was seen in the patient. Electrocardiogram (ECG) showed hyper acute ST elevation in anterolateral and inferior leads. He was a candidate for Primary percutaneous coronary intervention (PCI). Coronary angiography revealed left anterior descending artery (LAD) occlusion at far mid portion. After wiring with some difficulties regarding to thrombotic manifestation of the lesion, thrombosuction (with Export® AP aspiration catheter medtronic) performed which was unsuccessful and despite several efforts of balloon inflation by the mini trek RX 2*15mm coronary dilation catheter, Abbott vascular, the thrombolysis in myocardial infarction flow grade (TIMI) was 0-1. TIMI grade 0 reveals no penetration of contrast beyond stenosis (100% stenosis) and TIMI grade 1 reveals penetration of contrast beyond stenosis without perfusion of distal vessel (99% stenosis).

**Figure 1:**
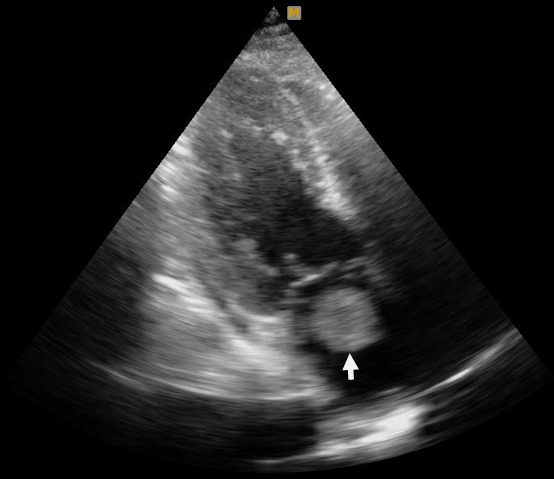
Echocardiography: left atrium myxoma

The procedure terminated unsuccessfully and the patient was admitted to cardiac care unit (CCU). His chest pain resolved 5 hours later and he was both hemodynamically and electrically stable. The day after admission echocardiography showed left ventricle ejection fraction (LVEF) 45% with some regional wall motion abnormalities in LAD territory and a large homogenous LA mass (3.5 × 3.5cm) attached to inter atrial septum that was protruded to mitral valve inflow (Figure 1). To prevent repeated embolization we decided to manage the patient surgically in an urgent setting. A large pedunculate mass, originated from left atrial septum covered with fresh clot was resected. Pathologic study showed a brown mucoid tissue which was compatible with cardiac myxoma. The patient had a normal post-operative period and discharged after 5 days.

## Discussion

The present case was an acute anterior ST elevation myocardial infarction (STEMI) with unsuccessful PCI and diagnosis of left atrial myxoma. Non-sclerotic coronary artery diseases (NA-CAD) have several etiologies: coronary vasospasm, Kounis syndrome and coronary embolisms [6]. Coronary vasospasm is defined as severe sudden vasoconstriction of epicardial coronary arteries which leads to stenosis or complete obstruction of the arteries [7]. Kounis syndrome is defined as acute coronary syndrome (symptoms such as chest pain relating to reduced blood flow to the heart) caused by an allergic reaction or a strong immune reaction to a drug or other substance. It was first described by Kounis and Zavras in 1991. The main pathophysiological mechanism of the allergic angina syndromes is the inflammatory mediators released during a hypersensitivity reaction triggered by allergens [8, 9]. Coronary embolisms are the rare causes of non-sclerotic coronary artery diseases.

Data on this area is only based on case reports or case reviews [10]. Coronary embolisms usually occur in the event of complete obstruction of the epicardial coronary arteries. Although its initial treatment is similar to patients with acute coronary syndrome (ACS), final management depends on the underlying conditions of the thrombus production. Coronary artery embolisms commonly associated with synthetic valve hypercoagulopathy [10], atrial fibrillation, endocarditis [11], cardiomyopathy [12], aortic valve thrombosis [13], foramen ovalve with paradoxical embolism [14] and even benign and malignant cardiac tumors [6]. Primary cardiac tumors are rare (0.0017% - 0.28%) and half of the benign heart tumors are myxomas. Systemic embolism is a well-known complication of myxoma, but coronary embolism is rare and about 0.06%. Among the right and left coronary artery, the right coronary artery embolism is more common than the left one.

Transthoracic echocardiography (TTE) is helpful in diagnosis of myxoma. Initial treatment depends on etiology and amount of the blockage. In some cases, thrombus aspiration is alone sufficient to re-establish the blood flow. In other cases balloonangioplasty or stent insertion is essential to access a TIMI grade 3. Underlying conditions of embolism should be treated uniquely. Anticoagulant treatments in cases of atrial fibrilation, synthetic valve thrombosis and intracardiac thrombosis recommended. Surgery interventions suggested as the best option for some cases including tumors. Embolism caused by myxoma is rare and manifestations are often cerebral. Since in patients with acute myocardial infarction reperfusion time is very decisive, echocardiography may not be the first therapeutic priority. Exactly, like this case that PCI was preceding the echocardiography. To improve prognosis and prevent embolism, surgery is the only treatment of atrial myxoma. Complete resection is recommended to prevent tumor recurrence.

## Conclusion

The present case was an acute anterior ST elevation myocardial infarction (STEMI) with unsuccessful PCI and diagnosis of left atrial myxoma. Non-sclerotic coronary artery diseases (NA-CAD) should be taken into account in young people with no atherosclerotic risk factors who refer to emergency with acute myocardial infarction.

## Competing interests

The authors declare no competing interests.
